# Supplementary material for: Molecular safeguarding of CRISPR gene drive experiments
Source: eLife. 2019 Jan 22;8:e41439. doi: 10.7554/eLife.41439 (PMC6358215; doi:10.7554/eLife.41439)
Supplement: Supplementary file 1. [file elife-41439-supp1.docx]

**SUPPLEMENTARY INFORMATION**

The following tables show the DNA fragments used for Gibson Assembly of each plasmid and the sequences of DNA oligos. PCR products are shown with the oligonucleotide primer pair used, and plasmid digests are shown with the restriction enzymes used.

| **p3xP3-dsRedv2** | *Template* | *Oligo/Enzyme 1* | *Oligo/Enzyme 2* |
| --- | --- | --- | --- |
| *PCR Product* | 3xP3-dsRed | pBB2_r_F | pBB2_r_R |
| *PCR Product* | g3xP3v2 | 3xP3v2_F | 3xP3v2_R |
| *PCR Product* | 3xP3-dsRed | dsRed_F | dsRed_R |
| *PCR Product* | pJFRC81 | P10_F | P10_R |

| **BHDgg1** | *Template* | *Oligo/Enzyme 1* | *Oligo/Enzyme 2* |
| --- | --- | --- | --- |
| *PCR Product* | none | GFPa_gRNA1_F | GFPa_gRNA1_R |
| *PCR Product* | pCFD3 | CFD_1_F | CFD_1_R |
| *PCR Product* | pCFD3 | CFD_2_F | CFD35_2_R |

| **BHDrg1** | *Template* | *Oligo/Enzyme 1* | *Oligo/Enzyme 2* |
| --- | --- | --- | --- |
| *PCR Product* | none | Rosy_gRNA1_F | Rosy_gRNA1_R |
| *PCR Product* | pCFD3 | CFD_1_F | CFD_1_R |
| *PCR Product* | pCFD3 | CFD_2_F | CFD35_2_R |

| **BHDaag1** | *Template* | *Oligo/Enzyme 1* | *Oligo/Enzyme 2* |
| --- | --- | --- | --- |
| *PCR Product* | none | Auto_gRNA1_F | Auto_gRNA1_R |
| *PCR Product* | pCFD3 | CFD_1_F | CFD_1_R |
| *PCR Product* | pCFD3 | CFD_2_F | CFD35_2_R |

| **BHDabg1** | *Template* | *Oligo/Enzyme 1* | *Oligo/Enzyme 2* |
| --- | --- | --- | --- |
| *PCR Product* | pCFD3 | CFD_1autoB_F | CFD_1_R |
| *PCR Product* | pCFD3 | CFD_2_F | CFD_2autoB_R |

| **BHDaeg1** | *Template* | *Oligo/Enzyme 1* | *Oligo/Enzyme 2* |
| --- | --- | --- | --- |
| *PCR Product* | none | AutoE_gRNA_F | AutoE_gRNA_R |
| *PCR Product* | pCFD3 | CFD_1_F | CFD_1_R |
| *PCR Product* | pCFD3 | CFD_2_F | CFD35_2_R |

| **BHDxyg1** | *Template* | *Oligo/Enzyme 1* | *Oligo/Enzyme 2* |
| --- | --- | --- | --- |
| *PCR Product* | none | Xy_gRNA_F | Xy_gRNA_R |
| *PCR Product* | pCFD3 | CFD_1_F | CFD_1_R |
| *PCR Product* | pCFD3 | CFD_2_F | CFD35_2_R |

| **BHDrN1i1** | *Template* | *Oligo/Enzyme 1* | *Oligo/Enzyme 2* |
| --- | --- | --- | --- |
| *PCR Product* | Genomic | RosyLeftN_F | RosyLeftN_R |
| *Plasmid Digest* | IHDyN1 | AvrII | SphI |

| **BHDaai1** | *Template* | *Oligo/Enzyme 1* | *Oligo/Enzyme 2* |
| --- | --- | --- | --- |
| *PCR Product* | Genomic DNA | AutoLeft_F | AutoLeft_R |
| *Plasmid Digest* | 3xP3-EGFP | XmaI | EcoRI |

| **BHDgN1ai1** | *Template* | *Oligo/Enzyme 1* | *Oligo/Enzyme 2* |
| --- | --- | --- | --- |
| *PCR Product* | BHDaa | GFPaLeft_F | GFPaLeft_R |
| *Plasmid Digest* | BHDrN1 | KpnI | XbaI |

| **BHDgN1bi1** | *Template* | *Oligo/Enzyme 1* | *Oligo/Enzyme 2* |
| --- | --- | --- | --- |
| *PCR Product* | ATSabG | EGFPabLeft_F | EGFPabLeft_R |
| *PCR Product* | BHDgN1a | Cas9Nos3b_F | Cas9Nos3_R |
| *Plasmid Digest* | BHDgN1a | KpnI | AscI |

| **BHDgN1ei1** | *Template* | *Oligo/Enzyme 1* | *Oligo/Enzyme 2* |
| --- | --- | --- | --- |
| *PCR Product* | ATSaeG | EGFPaeLeft_F | EGFPgLeft_R |
| *Plasmid Digest* | BHDgN1bv2 | KpnI | NheI |

| **BHDgN1yi1** | *Template* | *Oligo/Enzyme 1* | *Oligo/Enzyme 2* |
| --- | --- | --- | --- |
| *PCR Product* | ATSxyG | EGFPxyLeft_F | EGFPg2Left_R |
| *Plasmid Digest* | BHDgN1bv2 | KpnI | NheI |

| **ATSaaG** | *Template* | *Oligo/Enzyme 1* | *Oligo/Enzyme 2* |
| --- | --- | --- | --- |
| *PCR Product* | Genomic DNA | AutoRight_F | AutoRight_R |
| *Plasmid Digest* | BHDaai1 | SpeI | PstI |

| **ATSabG** | *Template* | *Oligo/Enzyme 1* | *Oligo/Enzyme 2* |
| --- | --- | --- | --- |
| *PCR Product* | 3xP3-EGFP | pBB2_b_F | pBB2_b_R |
| *PCR Product* | Genomic | AutoB_Left_F | AutoB_Left_R |
| *PCR Product* | 3xP3-EGFP | EGFP_b_F | EGFP_b_R |
| *PCR Product* | Genomic | AutoB_Right_F | AutoB_Right_R |

| **ATSaeG** | *Template* | *Oligo/Enzyme 1* | *Oligo/Enzyme 2* |
| --- | --- | --- | --- |
| *PCR Product* | 3xP3-EGFP | pBB2_e_F | pBB2_e_R |
| *PCR Product* | Genomic | AutoE_Left_F | AutoE_Left_R |
| *PCR Product* | 3xP3-EGFP | EGFP_e_F | EGFP_e_R |
| *PCR Product* | Genomic | AutoE_Right_F | AutoE_Right_R |

| **ATSxyG** | *Template* | *Oligo/Enzyme 1* | *Oligo/Enzyme 2* |
| --- | --- | --- | --- |
| *PCR Product* | 3xP3-EGFP | pBB2_y_F | pBB2_y_R |
| *PCR Product* | Genomic | Xy_Left_F | Xy_Left_R |
| *PCR Product* | 3xP3-EGFP | EGFP_y_F | EGFP_y_R |
| *PCR Product* | Genomic | Xy_Right_F | Xy_Right_R |

| **BHDaaN** | *Template* | *Oligo/Enzyme 1* | *Oligo/Enzyme 2* |
| --- | --- | --- | --- |
| *PCR Product* | pnos-Cas9-nos | NosCas9_1b_F | NosCas9_1_R |
| *PCR Product* | pnos-Cas9-nos | NosCas9_2_F | NosCas9_2b_R |
| *Plasmid Digest* | BHDaa | SphI | NotI |

| **BHDrN1** | *Template* | *Oligo/Enzyme 1* | *Oligo/Enzyme 2* |
| --- | --- | --- | --- |
| *PCR Product* | BHDrg1 | U6_3_gRNA1_F | Rosy_U6_3_gRNA1_R |
| *PCR Product* | Genomic DNA | RosyRight1_F | RosyRight1N_R |
| *Plasmid Digest* | BHDrNi1 | SpeI | DraIII |

| **BHDgN1a** | *Template* | *Oligo/Enzyme 1* | *Oligo/Enzyme 2* |
| --- | --- | --- | --- |
| *PCR Product* | BHDgg1 | U6_3_gRNA1_v2_F | EGFPa_U6_3_gRNA1_R |
| *PCR Product* | BHDaa | EGFPaRight_F | EGFPaRight_R |
| *Plasmid Digest* | BHDgN1ai1 | SpeI | DraIII |

| **BHDgN1b** | *Template* | *Oligo/Enzyme 1* | *Oligo/Enzyme 2* |
| --- | --- | --- | --- |
| *PCR Product* | BHDgg1 | U6_3_gRNA1_v3_F | EGFPb_U6_3_gRNA1_R |
| *PCR Product* | ATSabG | EGFPabRight_F | EGFPabRight_R |
| *Plasmid Digest* | BHDgN1bi1 | AgeI | DraIII |

| **BHDgN1bv2** | *Template* | *Oligo/Enzyme 1* | *Oligo/Enzyme 2* |
| --- | --- | --- | --- |
| *PCR Product* | p3xP3-dsRedv2 | b1v2_F | b1v2_R |
| *Plasmid Digest* | BHDgN1b | NotI | MluI |

| **BHDgN1e** | *Template* | *Oligo/Enzyme 1* | *Oligo/Enzyme 2* |
| --- | --- | --- | --- |
| *PCR Product* | ATSaeG | EGFPgRight_F | EGFPaeRight_R |
| *Plasmid Digest* | BHDgN1ei1 | XbaI | DraIII |

| **BHDgN1e** | *Template* | *Oligo/Enzyme 1* | *Oligo/Enzyme 2* |
| --- | --- | --- | --- |
| *PCR Product* | ATSxyG | EGFPgRight_F | EGFPxyRight_R |
| *Plasmid Digest* | BHDgN1yi1 | XbaI | DraIII |

**Construction primers**

3xP3v2_F: ATTCTGAACATTATCGCGAGCCGGATCTAATTC

3xP3v2_R: CCTCGGAGGAGGCCATGCCTCGCTGCGGCTTC

Auto_gRNA1_F: TATATATAGACCTATTTTCAATTTAACGTCGAATCGATTAGCATCAACCA

Auto_gRNA1_R: ATTTTAACTTGCTATTTCTAGCTCTAAAACTGGTTGATGCTAATCGATTC

AutoB_Left_F: ACATTATCGCGAGCCCCCACATTCTCTCATCGCAGG

AutoB_Left_R: ATTAGATCCCGTACGGTGCGGATGCTGTTTGTTTGT

AutoB_Right_F: ATGTATCTTAACCGGAGGTTTCAAAGTGCAGCGATAAGA

AutoB_Right_R: CAGAAGGCCCCTGACATGAAGGTGGTCTCCAACGC

AutoE_gRNA_F: TATATATAGACCTATTTTCAATTTAACGTCGCAATTTAAAAGACAGCCGG

AutoE_gRNA_R: ATTTTAACTTGCTATTTCTAGCTCTAAAACCCGGCTGTCTTTTAAATTGC

AutoE_Left_F: ACATTATCGCGAGCCGAGTCCGGCCCAGGAATCGTC

AutoE_Left_R: ATTAGATCCCGTACGCGGCGGTCGCTTCTCCCA

AutoE_Right_F: ATGTATCTTAACCGGGCTGTCTTTTAAATTGCAGTGCC

AutoE_Right_R: CAGAAGGCCCCTGACCAGCAAAGACATGGTATCGCC

AutoLeft_F: AAGGCTCAGTCGAAAGACTGGGCCTTTCGCCCGGTTGCGAACAAAACGCCCATCGAGGA

AutoLeft_R: TGCATATGTCCGCGGCCGCTAGCATGCAAGAATTACCAGGGCTGCAAAGAGCGGG

AutoRight_F: ATGCTATACGAAGTTATAGAAGAGCACTAGTTGATGCTAATCGATTCAATTCTGTAATGC

AutoRight_R: TTGAACTCGATTGACGGAAGAGCCTCGAGCTGCATATGACGAAATCAAGGCTAAGGTCG

b1v2_F: ATTTCGAGGTTAAAACGGTCGAAGCGCGGCCGCGGATCTAATTCAATTAGAGACTAATTC

b1v2_R: GAGTAGGAGCAATCACAGGTGAGCAAAAAAACGCGTGTTAACTCGAATCGCTATCCA

Cas9Nos3_R: TATCCACTTGTTTACTCTGACCAACT

Cas9Nos3b_F: AGCTGACCCTGAGCTAGCTCCTTCCTGGCCCTTTTCGAG

CFD_1_F: GTTTTAGAGCTAGAAATAGCAAGTTAAAATAAGG

CFD_1_R: GGCTATGCGTTGTTTGTTCTGC

CFD_1autoB_F: ACGTCGCTGCACTTTGAAACCTGTGGTTTTAGAGCTAGAAATAGCAAGTTAAAATAAGG

CFD_2_F: AACAGTAGGCAGAACAAACAACGC

CFD_2autoB_R: AAAACCACAGGTTTCAAAGTGCAGCGACGTTAAATTGAAAATAGGTCTATATATACGAA

CFD35_2_R: CGACGTTAAATTGAAAATAGGTCTATATATACG

dsRed_F: AGCCGCAGCGAGGCATGGCCTCCTCCGAGGACGT

dsRed_R: TTAAAAACGATTCATCTACAGGAACAGGTGGTGGCGG

EGFP_b_F: AAACAGCATCCGCACCGTACGGGATCTAATTCAATTAGAGACTAA

EGFP_b_R: TGCACTTTGAAACCTCCGGTTAAGATACATTGATGAGTTTGG

EGFP_e_F: GAGAAGCGACCGCCGCGTACGGGATCTAATTCAATTAGAGACTAA

EGFP_e_R: AATTTAAAAGACAGCCCGGTTAAGATACATTGATGAGTTTGG

EGFP_y_F: TACAATTTTCCTAATCGTACGGGATCTAATTCAATTAGAGACTAA

EGFP_y_R: AATACAAGTAAGGAACCGGTTAAGATACATTGATGAGTTTGG

EGFPa_U6_3_gRNA1_R: TGGTGCAGATGAACTATGCATACGCATTAAGCGAACA

EGFPabLeft_F: ATTAACCAATTCTGAACATTATCGCCTAGGGTACCCCCACATTCTCTCATCGCAGG

EGFPabLeft_R: GGCCAGGAAGGAGCTAGCTCAGGGTCAGCTTGCCGTA

EGFPabRight_F: ATGCGTATGCATTCTAGAAGTTCATCTGCACCACCGGC

EGFPabRight_R: TGATTGACGGAAGAGCCTCGAGCTGCACACACAGTGATGAAGGTGGTCTCCAACGCCT

EGFPaeLeft_F: ATTAACCAATTCTGAACATTATCGCCTAGGGTACCGAGTCCGGCCCAGGAATCGTC

EGFPaeRight_R: TGATTGACGGAAGAGCCTCGAGCTGCACACACAGTGCAGCAAAGACATGGTATCGCC

EGFPaRight_F: TTAATGCGTATGCATAGTTCATCTGCACCACCGGCA

EGFPaRight_R: GACGGAAGAGCCTCGAGCTGCACACACAGTGTGAGCATAACCAAGGTCACGAAAGGT

EGFPb_U6_3_gRNA1_R: TGCAGATGAACTTCTAGAATGCATACGCATTAAGCGAACA

EGFPg2Left_R: CGGCGTTTCTCGAAAAGGGCCAGGAAGGAGCTAGCTCAGGGTCAGCTTGCCGTAG

EGFPgLeft_R: CGGCGTTTCTCGAAAAGGGCCAGGAAGGAGCTAGCTCAGGGTCAGCTTGCCGTAGGT

EGFPgRight_F: TTTTTAATGTTCGCTTAATGCGTATGCATTCTAGAAGTTCATCTGCACCACCGG

EGFPxyLeft_F: TAACCAATTCTGAACATTATCGCCTAGGGTACCTTTCTTGCTGTCTGACTTGGATTATTC

EGFPxyRight_R: GATTGACGGAAGAGCCTCGAGCTGCACACACAGTGCAAATTAGCCACACGTAGGATATTC

GFPa_gRNA1_F: TATATATAGACCTATTTTCAATTTAACGTCGGTGGTGCAGATGAACTTCA

GFPa_gRNA1_R: ATTTTAACTTGCTATTTCTAGCTCTAAAACTGAAGTTCATCTGCACCACC

GFPaLeft_F: ATTAACCAATTCTGAACATTATCGCCTAGGGTACCGCGCAGCGCCAATGATAGGTACAAT

GFPaLeft_R: TTTCTCGAAAAGGGCCAGGAAGGAGCATGTCTAGATCAGGGTCAGCTTGCCGTAGGTG

P10_F: CACCTGTTCCTGTAGATGAATCGTTTTTAAAATAACAAATCAATT

P10_R: CAGAAGGCCCCTGACGTTAACTCGAATCGCTATCCA

pBB2_b_F: GGAGACCACCTTCATGTCAGGGGCCTTCTGCTTAGT

pBB2_b_R: ATGAGAGAATGTGGGGGCTCGCGATAATGTTCAGAATTG

pBB2_e_F: ACCATGTCTTTGCTGGTCAGGGGCCTTCTGCTTAGT

pBB2_e_R: TCCTGGGCCGGACTCGGCTCGCGATAATGTTCAGAATTG

pBB2_r_F: GCGATTCGAGTTAACGTCAGGGGCCTTCTGCTTAGT

pBB2_r_R: TTAGATCCGGCTCGCGATAATGTTCAGAATTG

pBB2_y_F: CGTGTGGCTAATTTGGTCAGGGGCCTTCTGCTTAGT

pBB2_y_R: TCAGACAGCAAGAAAGGCTCGCGATAATGTTCAGAATTG

Rosy_gRNA1_F: TATATATAGACCTATTTTCAATTTAACGTCGATATGCTTCACTACTTTGC

Rosy_gRNA1_R: ATTTTAACTTGCTATTTCTAGCTCTAAAACGCAAAGTAGTGAAGCATATC

Rosy_U6_3_gRNA1_R: TGCCGGCAACGCGTCCTGCAGGATGCATACGCATTAAGCGAACATT

RosyLeftN_F: ATTAACCAATTCTGAACATTATCGCCTAGGGTACCTTATTCCAGGTGACCGAAGTGTCG

RosyLeftN_R: TTTCTCGAAAAGGGCCAGGAAGGAGCATGTCTAGAAAGTAGTGAAGCATATCCACGGTGC

RosyRight1_F: GTATGCATCCTGCAGGACGCGTTGCCGGCAAGCAGATCCGCAA

RosyRight1N_R: GATTGACGGAAGAGCCTCGAGCTGCACACACAGTGTGGCGCCCACTATCTGACCATAGCA

U6_3_gRNA1_F: ATGCTATACGAAGTTATAGAAGAGCACTAGGCTAGCTTTTTTGCTCACCTGTGATTGCTC

U6_3_gRNA1_v2_F: GTATGCTATACGAAGTTATAGAAGAGCACTAGTTTTTTTGCTCACCTGTGATTGCTC

U6_3_gRNA1_v3_F: GTCCAAACTCATCAATGTATCTTAACCGGTACGCGTTTTTTTGCTCACCTGTGATTGCTC

Xy_gRNA_F: TATATATAGACCTATTTTCAATTTAACGTCGCAATACAAGTAAGGAAATTG

Xy_gRNA_R: ATTTTAACTTGCTATTTCTAGCTCTAAAACAATTTCCTTACTTGTATTGCG

Xy_Left_F: ACATTATCGCGAGCCTTTCTTGCTGTCTGACTTGGA

Xy_Left_R: ATTAGATCCCGTACGATTAGGAAAATTGTAAAAAAAACACGATG

Xy_Right_F: ATGTATCTTAACCGGTTCCTTACTTGTATTGCCTACTTT

Xy_Right_R: CAGAAGGCCCCTGACCAAATTAGCCACACGTAGGAT

**Sequencing primers**

AutoB_Left_S_F: ATGACCTTTCGTGACCTTGG

AutoB_Left_S_R: ACGACCAATTGAACTCAGACC

AutoB_Right_S_F: ACATCGTTCCTTTGGGATTG

AutoB_Right_S_R: AAAACTTGTGCATCGAATGAAG

AutoE_Left_S_F: GTGACTTTGAATGTCAGAATATCAACT

AutoE_Left_S_R: CGGCCATCTCACACATTGTTAC

AutoE_Right_S_F: GTACATTTGTGTGGCTGGTCC

AutoE_Right_S_R: TCGTCTATTTTCGATTTGGGCAC

AutoLeft_S_F: GCAGCGCCAATGATAGGTACA

AutoLeft_S_R: TTGCATGTCGGCATACTTCGG

AutoRight_S_F: TGCATATCTCAGCAAGTCCGC

AutoRight_S_R: GTTGAATAAAGTATCGCCCTGTTAC

EGFP_S_F: AGCGCACCATCTTCTTCAAGG

EGFP_S_R: AGTTGTACTCCAGCTTGTGCC

EGFPaLeft_S_F: ATCGATTTCGAACCCTCGACC

EGFPaLeft_S_R: GCTTGTTTATTTGCTTAGCTTTCGC

EGFPaRight_S_F: CCAGGAGCGCACCATCTTCTT

EGFPaRight_S_R: GTCCTCCTTGAAGTCGATGCC

Xy_Left_S_F: GGGTTTCGCATCTTCTGGATC

Xy_Left_S_R: GCTCTGACAACCCCAACAGAA

Xy_Right_S_F: TGCATACTCTTGGTCACAGCA

Xy_Right_S_R: AGCTCTGTTTCAAGAACATTTCCG

**gBlock**

g3xP3v2:

CATTATCGCGAGCCGGATCTAATTCAATTAGAGACTAATTCAATTAGAGCTAATTCAATTAGGCTAAGCCGTGTATAGAGAACAACCACACCCCGCCGGAGTATAAATAGAGGCGCTTCGTCTACGGAGCGACAATTTAATTCAAACAAGCAAAGTGAACACGTCGCTAAGCGAAAGCTAAGCAAATAAACAAGCGCAGCTGAACAAGCTAAACAATCGGTCCGAAGCCGCAGCGAGGCAT
